# Weight associated factors in relation to health-related quality of life (HRQoL) in Iranian adolescents

**DOI:** 10.1186/s12955-018-1074-9

**Published:** 2019-01-07

**Authors:** Sara Jalali-Farahani, Behnood Abbasi, Mahdis Daniali

**Affiliations:** 1 Faculty of Health, Saveh University of Medical Sciences, Saveh, Iran; 2 Department of Nutrition, Science and Research Branch, Islamic Azad University, Tehran, Iran; 3 School of Public Health, University of Queensland, Brisbane, Queensland Australia

**Keywords:** Health-related quality of life, Overweight, Obesity, Weight perception, Adolescents

## Abstract

**Objective:**

This study aimed to explore body weight factors associated with HRQoL of Iranian adolescents.

**Methods:**

A total of 584 adolescents (12–18 years) were included in this cross-sectional study. Participants were selected using a multi-stage sampling method from schools located in Isfahan city in Iran. Body weight, height and waist circumference of adolescents were measured according to standard protocol and body mass index-for-age was determined using Anthroplus software. Adolescents completed a set of self-administered questionnaires which included questions about perceptions of adolescents regarding their body weight status and adolescents’ satisfaction regarding their body weight. To assess HRQoL, adolescents completed the Iranian version of the Pediatric Quality of Life Inventory™ version 4.0 (PedsQL™ 4.0).

**Results:**

The mean age of participants was 15.1 ± 1.3 years. The overall prevalence of overweight and obesity were higher in boys compared to girls (34.5 vs. 26.7% respectively). More boys (49.5%) had incorrect perceptions regarding their body weight status compared to girls (37.0%), and more girls (39.0%) were dissatisfied with their body weight compared to boys (28.8%). HRQoL scores were not significantly different among body weight status groups except for significantly lower physical functioning scores in underweight adolescents compared to their counterparts (*p* = 0.049; power = 64%). Based on perception of body weight status, only boys who perceived themselves as underweight or overweight reported lower scores in different subscales of HRQoL compared to those who perceived themselves as normal weight (*p* < 0.05; power > 85%). In girls, body weight dissatisfaction was significantly associated with poorer HRQoL in psychosocial dimensions, while in boys, body weight dissatisfaction was significantly associated with poorer HRQoL in physical and social functioning subscales (*p* < 0.05; power > 85%).

**Conclusion:**

In conclusion, impairment in HRQoL mainly depends on adolescents’ perception and dissatisfaction regarding their body weight and less on their actual body weight status. These findings implying that more attention should be paid to body image as an important target for interventions aiming at promoting HRQoL during early years of life.

## Introduction

Health-related quality of life (HRQoL) is a fundamental concept which has become a popular area of health research in recent years. HRQoL evaluates the self-perceived health of an individual in three main dimensions of health (physical, mental and social well-being), as delineated by the World Health Organization’s (WHO) definition for health (World Health Organization, 1948). Previous studies have found several factors to be associated with HRQoL in adolescents, including socio-demographic factors, sex and age [1, 2]. Furthermore, several chronic diseases were shown to influence impairment of HRQoL in adolescents [3]. Although many people do not perceive obesity and overweight as illnesses, they have been found to be associated with low self-esteem, depression, stigmatization, social marginalization, impaired physical health and higher incidence of diseases, all of which may influence the HRQoL of adolescents [4, 5]. In this context, a number of previous studies in both clinical and community settings reported impairments in different dimensions of HRQoL in adolescents who are overweight and obese compared to their normal weight counterparts [6–8]. However, this association was not observed in some previous studies [9–11] in which there was an inconsistency regarding the association of overweight/obesity and HRQoL in adolescents.

This inconsistency in previous findings may be due to influencing factors such as body dissatisfaction and adolescents’ perception regarding their weight status as prescribed by their cultural context and value system. Evidence indicates that body dissatisfaction mediates the impact of obesity on low self-esteem and high depressive mood [12]. Given the role of body dissatisfaction in the mental well-being of adolescents, body dissatisfaction may be responsible for the impaired HRQoL in adolescents who are overweight and obese. In this context, previous studies reported that the association between body weight and HRQoL was mediated by body dissatisfaction [13]. In addition, there is evidence indicating that HRQoL in adolescents who are obese was varied by weight perception rather than actual body weight [14] and adolescents who were actually obese reported better HRQoL compared to those who perceived themselves as overweight [15]. Therefore, body dissatisfaction and perceived weight status are factors which may potentially negatively impact HRQoL in adolescents. Body dissatisfaction commonly has been linked to obesity, especially in girls and women; however, Richards et al. and Presnell et al. found underweight to be associated with body dissatisfaction. They reported that boys were less satisfied when they were either underweight or overweight [16, 17]. Research in the past decade revealed that perception of weight may differ in adolescent boys and girls. While adolescent girls desire to be thinner, boys tend to be muscular and heavier and consequently boys are less likely than girls to consider themselves overweight [18–20].

Past studies conducted among Iranian adolescents were mainly focused on actual body weight [21, 22] and few studies investigated the association of perceived weight status and body dissatisfaction with HRQoL [23]. Considering the lack of evidence regarding the association of body weights status, weight perception and body dissatisfaction with HRQoL in Iranian adolescents, this study was conducted to investigate the factors associated with HRQoL of Iranian adolescents residing in Isfahan.

## Method

### Participants

In this cross-sectional study, participants were selected from schools in Isfahan city, the third largest city located in central Iran. Multi-stage sampling method was used for the selection of study participants aged 12–18 years old across schools in Isfahan. First, Isfahan city was divided into two geographical zones (North and South). Then, in each geographical zone, one district was randomly selected of which a total of four schools were randomly selected. A total of 648 adolescents were randomly selected across all selected schools and were invited to participate in the study. Eligible participants were those between the ages of 12 and 18 who consented to participation, submitted parental permission forms, and did not have any chronic diseases.

Prior to data collection, ethics approval (ethical approval number: Ir.savehums.rec.1395.29) was obtained from the Ethics Committee of Saveh University of Medical Sciences in Iran. Furthermore, approvals were obtained from the Ministry of Education and selected schools. In addition, both adolescents and their parents who agreed to participate in the study were asked to sign written informed consent forms. After explaining the study aims and its procedure to the adolescents, the parental forms including parental consent forms, information sheets and a parental questionnaire were distributed to the adolescents. Adolescents were asked to share the forms with their parents and bring back the completed forms the next day.

During the data collection process, adolescents who provided their signed informed consent forms to participate in the study were invited for anthropometric measurements by trained research assistants. Then, after a brief explanation on how to complete the questionnaires, participants completed the adolescent questionnaires (including self-report of HRQoL and questions about perceptions and satisfactions regarding their body weight status). Of 648 adolescents who were invited to participate in this study, 41 adolescents were excluded as they did not return parental forms or they refused to participate in the study, and 23 adolescents were excluded due to having chronic diseases. Data from 584 adolescents was analyzed.

### Measurements

Trained research assistants measured body weight, height and waist circumference of the students according to standard protocol. Body weight of participants was measured using the Beurer Weighing Scale and recorded to the nearest 0.1 kg. Body height was measured using the Seca Body Meter and recorded to the nearest 0.1 cm. Waist circumference was measured according to the WHO protocol [24] at the midpoint between the lowest rib and the top of iliac crest, using a tape measure and recorded to the nearest 0.1 cm. Body mass index-for-age (BMI Z score) was determined using the Anthroplus software and body weight status of students was determined according to the World Health Organization (WHO) growth reference for 5–19 years old children.

Health-related quality of life was assessed using the Pediatric Quality of Life Inventory™ version 4.0 (PedsQL™ 4.0) [25]. This self-administered questionnaire has 23 items and consists of four subscales: physical functioning, emotional functioning, social functioning and school functioning. The respondent chooses his/her answer from five response choices ranging from 0 = never a problem to 4 = almost always a problem. To calculate the scores, the 0–4 scale items were transformed to 0–100 as follows: 0 = 100, 1 = 75, 2 = 50, 3 = 25, 4 = 0; therefore, a higher score indicates better HRQoL. In the current study, the Iranian version of PedsQL™ 4.0 was used; the high validity and reliability of Iranian version has been reported previously [26].

To obtain participant perception regarding their body weight status, adolescents were asked to answer a simple question: “What do you think about your current body weight status?” The adolescents chose their answer from five choices including “severe thinness”, “thinness”, “normal weight”, “overweight” and “obese”. The participant’s perception regarding their body weight status was compared with their actual body weight status. The results of this comparison were categorized into three groups including under-estimation, correct-estimation and over-estimation. When participant’s perception regarding their body weight status was similar to their actual body weight status, the participant was categorized in the correct-estimation group. When participants answered a lower or higher body weight status group compared to their actual body weight status, they were placed into under-estimation or over-estimation groups, respectively. To obtain participants’ satisfaction regarding their body weight, adolescents were asked to respond to another simple question: “How satisfied are you with your current body weight?” The adolescents chose their answer from five choices including “very satisfied”, “fairly satisfied”, “neither satisfied nor dissatisfied”, “fairly dissatisfied” and “very dissatisfied”. Their responses were categorized into three groups: satisfied (including both “very satisfied” and “fairly satisfied”), neither satisfied nor dissatisfied, and dissatisfied (including both “very dissatisfied” and “fairly dissatisfied”).

### Statistical analysis

Data analysis was conducted using SPSS software (version 20.0). For continues variables, means and standard deviations and for categorical variables frequencies (percentages) were reported as descriptive statistics. The distribution of categorical variables were compared between genders using the Chi-Square test. To determine the association between BMI Z score and HRQoL scores as well as waist circumference and HRQoL scores Spearman’s correlation test was conducted. To compare mean subscales and total scores of HRQoL between the two groups, independent t-test analysis was used. The general linear model was used to compare subscales and total scores of HRQoL among different groups of independent variables including adolescents’ body weight status, adolescents’ perception regarding their body weight status and adolescents’ satisfaction regarding their body weight. If significant differences existed, *Bonferroni* Post hoc test was conducted to determine which two groups were significantly different in HRQoL scores. In all models, HRQoL score was considered a dependent variables and effects of parental educational level (Less than high school diploma/High school diploma/University degree), maternal working status (Housewife/Working outside the home) and paternal working status (Self-employed/Employee/Laborer/Retired/Jobless) were adjusted. In each model, a two-way interaction of independent variables (adolescents’ body weight status, adolescents’ perception regarding their body weight status and adolescents’ satisfaction regarding their body weight) and sex was tested. Where interaction effects were significant, sex-specific analysis was conducted. A significant level of 0.05 was utilized.

## Results

Table 1 indicates the descriptive statistics of study participants. Mean age, BMI Z score and waist circumference of participants were 15.1 ± 1.3 years, 0.35 ± 1.27 and 75.3 ± 10.4 cm, respectively. Boys had significantly higher BMI Z scores compared to girls (*t* = 2.15, *p* = 0.032) while mean of waist circumference was significantly higher in girls compared to boys (*t* = − 2.45, *p* = 0.015). The prevalence of underweight, normal weight, overweight and obesity was significantly different among boys and girls (χ^2^ = 12.08, *p* = 0.007). About two thirds of participants (66.6%) had normal weight and 3.1% of participants were underweight. The overall prevalence of overweight and obesity was higher in boys compared to girls (34.5 vs. 26.7% respectively). Adolescents’ perceptions regarding their body weight status were not significantly different in girls and boys. According to adolescents’ perceptions regarding their body weight status, one in five of them classified themselves as underweight (21.8%), half of them perceived that they have normal weight and 23.4 and 3.1% of them perceived that they are overweight and obese respectively. More boys (49.5%) had incorrect perceptions regarding their body weight status compared to girls (37.0%). Adolescents’ satisfaction regarding their body weight were significantly different between boys and girls (χ^2^ = 12.25, *p* = 0.002). More girls (39.0%) were dissatisfied with their body weight compared to boys (28.8%). Information about socio-demographic characteristics of participants are also presented in Table 1.

**Table 1:** Descriptive statistics of participants

	Girls (*n* = 308)	Boys (*n* = 276)	Total (*n* = 584)
Age (year)^a^	15.1 ± 1.4	15.2 ± 1.1	15.1 ± 1.3
BMI (kg/m^2^)^a^	21.5 ± 3.9	22.1 ± 4.6	21.8 ± 4.3
BMI Z score^a^	0.24 ± 1.17	0.47 ± 1.36	0.35 ± 1.27
Waist circumference (cm)^a^	76.3 ± 9.6	74.2 ± 11.1	75.3 ± 10.4
Adolescents’ body weight status^b^			
-Underweight	11(3.6%)	7(2.5%)	18(3.1%)
-Normal weight	215(69.8%)	174(63.0%)	389(66.6%)
-Overweight	59(19.2%)	49(17.8%)	108(18.5%)
-Obese	23(7.5%)	46(16.7%)	69(11.8%)
Adolescents’ perceptions regarding their body weight status^b^ (*n* = 578)			
-Underweight	59(19.5%)	67(24.4%)	126(21.8%)
-Normal weight	159(52.5%)	140(50.9%)	299(51.7%)
-Overweight	79(26.1%)	56(20.4%)	135(23.4%)
-Obese	6(2.0%)	12(4.4%)	18(3.1%)
Adolescents’ satisfaction regarding their body weight^b^ (*n* = 575)			
- Satisfied	155(51.6%)	181(65.8%)	336(58.4%)
-In between satisfied and dissatisfied	28(9.3%)	15(5.5%)	43(7.5%)
- Dissatisfied	117(39.0%)	79(28.8%)	196(34.1%)
Marital status of parents^b^(*n* = 582)			
-Married	287 (93.8%)	268 (97.1%)	555(95.4%)
-Single parent (Divorced/Widowed)	19 (6.2%)	8 (2.9%)	27 (4.6%)
Mothers’ education^b^ (*n* = 583)			
- Less than high school diploma	91 (29.6%)	92 (33.3%)	183 (31.4%)
- High school diploma	143 (46.6%)	98 (35.5%)	241 (41.3%)
- University degree	73 (23.8%)	86 (31.2%)	159 (27.3%)
Mothers’ working status^b^(*n* = 581)			
- Housewife	246 (80.1%)	210 (76.6%)	456 (78.5%)
- Working	61 (19.9%)	64 (23.4%)	125 (21.5%)
Fathers’ education^b^(*n* = 574)			
- Less than high school diploma	85 (28.3%)	76 (27.8%)	161 (28.1%)
- High school diploma	131 (43.7%)	89 (32.5%)	220 (38.3%)
- University degree	84 (28.0%)	109 (39.8%)	193 (33.5%)
Father’s job^b^(n = 574)			
- Self employed	140 (46.7%)	123 (44.9%)	263 (45.8%)
- Employee	95 (31.7%)	93 (33.9%)	188 (32.8%)
- Laborer	26 (8.7%)	23 (8.4%)	49 (8.5%)
- Retired	31 (10.3%)	30 (10.9%)	61 (10.6%)
- Jobless	8 (2.7%)	5 (1.8%)	13 (2.3%)

^a^Data have been reported as mean ± sd. ^b^Data have been reported as frequency(percentage)

Means of subscales and total scores of self-reported HRQoL are presented in Table 2. As it is indicated in both sexes, the highest scores were in social functioning and the lowest scores were in emotional functioning. There was a significant difference in HRQoL scores between boys and girls in physical functioning and emotional functioning subscales, in which boys scored significantly higher.

**Table 2:** Adolescent self-reported HRQoL scores by sex groups

	Adolescent self-report			t value	*p*-value
	Total	Girls	Boys		
Physical functioning	79.0 ± 14.1	75.4 ± 13.9	83.1 ± 13.1	6.79	< 0.001
Emotional functioning	64.3 ± 20.2	59.9 ± 20.2	68.9 ± 19.2	5.47	< 0.001
Social functioning	82.1 ± 15.7	81.7 ± 15.5	82.6 ± 15.9	0.67	0.501
School functioning	71.3 ± 17.6	70.6 ± 17.2	71.9 ± 18.2	0.91	0.363
HRQOL total score	74.8 ± 12.6	72.4 ± 12.7	77.5 ± 11.9	4.97	< 0.001

Note: Independent sample t test has been conducted. Data have been reported as mean ± sd

There were no significant associations between BMI Z scores and HRQoL scores in both boys and girls. In addition, waist circumference was not significantly correlated with HRQoL scores in boys; however, it had significant inverse correlation with social functioning scores in girls (r_s_ = − 0.15, *p* = 0.01). Using the general linear model (ANCOVA), adolescent self-reported HRQoL scores were compared among different groups of body weight status groups after adjusting for parental educational level, mother’s working status and father’s working status (Table 3). First, two-way interaction of adolescents’ body weight status*sex was tested and it was not significant (Physical functioning: F_3, 551_: 1.11, *p* = 0.345; Emotional functioning: F_3, 551_: 1.73, *p* = 0.160; Social functioning: F_3, 551_: 1.89, *p* = 0.130; School functioning: F_3, 551_: 0.39, *p* = 0.760; HRQoL total score: F_3, 551_: 1.26, *p* = 0.288). Thus, sex was adjusted in the model and subgroup analysis was not performed separately for boys and girls. As it is indicated in Table 3, except for physical functioning subscale, there was no significant difference in HRQoL scores among underweight, normal weight, overweight and obese adolescents. Results of the post hoc test showed that underweight adolescents had significantly lower physical functioning scores compared to their normal weight (*p* = 0.020), overweight (*p* = 0.014) and obese (*p* = 0.026) counterparts.

**Table 3:** Adolescent self-reported HRQoL scores in different body weight status groups

	Underweight	Normal weight	Overweight	Obese	F value	*p*-value	Effect size (power)*
Physical functioning	70.5 ± 14.0^a^	79.3 ± 23.7^b^	80.0 ± 16.6^b^	78.5 ± 14.9^b^	2.63	0.049	0.014(0.64)
Emotional functioning	65.2 ± 20.4	63.7 ± 33.5	63.4 ± 23.9	63.1 ± 22.4	0.06	0.982	0.00(0.06)
Social functioning	79.5 ± 16.1	83.3 ± 27.6	81.9 ± 19.7	78.0 ± 17.4	2.46	0.062	0.013(0.61)
School functioning	70.3 ± 18.7	71.7 ± 29.6	70.8 ± 21.8	71.9 ± 19.9	0.10	0.960	0.001(0.07)
HRQOL total score	71.2 ± 12.7	75.1 ± 21.7	74.8 ± 15.6	73.6 ± 14.1	0.82	0.482	0.004(0.23)

Note: Analysis of covariance (ANCOVA) test was conducted and adolescent’s sex, parental educational level, mother's working status and father’s job were adjusted in the model. Adjusted mean and standard deviation have been reported. ^a^ and ^b^: Results of Bonferroni’s post test have been indicated as superscript letters in each row. Means in a row without a common superscript letter significantly differ. * Partial Eta squared is reported as effect size

Using the general linear model (ANCOVA), adolescent self-reported HRQoL scores were compared among different groups of body weight status groups according to adolescents’ perception (Table 4). Primary findings of testing two-way interactions indicated that two-way interactions of adolescents’ perception regarding their body weight status*sex for most HRQoL scores were statistically significant (Physical functioning: F_6, 546_: 3.33, *p* = 0.003; Emotional functioning: F_6, 546_: 2.72, *p* = 0.013; Social functioning: F_6, 546_: 2.95, *p* = 0.008; School functioning: F_6, 546_: 1.45, *p* = 0.192; HRQoL total score: F_6, 546_: 4.16, *p* < 0.001); hence, subgroup analysis was performed separately in boys and girls. As indicated in Table 4, none of the subscales and total scores of HRQoL were significantly different among body weight status groups according to the perception of girls regarding their body weight status. However, physical functioning, emotional functioning, social functioning and total scores of HRQoL were significantly different among body weight status groups according to perception of boys regarding their body weight status groups. The results of *post hos* test indicated that based on boys’ perceptions, those boys who perceived themselves as underweight, had significantly lower scores in physical functioning (*p* = 0.001), emotional functioning (*p* < 0.001), social functioning (*p* = 0.009) and total HRQoL (*p* < 0.001), compared to those who perceived themselves as normal weight. In addition, those boys who perceived themselves as overweight had significantly lower scores in physical functioning (*p* = 0.015), social functioning (*p* = 0.033) and total HRQoL (*p* = 0.011) compared to boys who perceived themselves as normal weight.

**Table 4:** Adolescent self-reported HRQoL scores according to adolescents’ perception regarding their body weight status

	Underweight	Normal weight	Overweight	Obese	F value	*p*-value	Effect size (power)*
Girls							
Physical functioning	72.9 ± 17.6	75.3 ± 21.4	74.7 ± 17.7	84.9 ± 15.4	1.26	0.290	0.01(0.34)
Emotional functioning	60.0 ± 26.1	59.4 ± 31.5	56.6 ± 26.6	64.5 ± 22.8	0.51	0.674	0.01(0.15)
Social functioning	83.7 ± 19.2	84.4 ± 23.9	80.0 ± 19.5	81.7 ± 17.1	1.43	0.236	0.02(0.38)
School functioning	69.9 ± 21.5	71.5 ± 26.4	68.2 ± 22.2	74.5 ± 19.1	0.74	0.527	0.01(0.21)
HRQOL total score	71.8 ± 16.1	73.0 ± 20.1	70.5 ± 16.0	77.5 ± 13.9	0.98	0.403	0.01(0.27)
Boys							
Physical functioning	79.1 ± 17.2^ac^	86.1 ± 21.3^b^	79.9 ± 16.4^c^	84.0 ± 13.8^abc^	5.62	0.001	0.06(0.94)
Emotional functioning	61.2 ± 34.3^a^	71.5 ± 29.5^b^	65.9 ± 22.4^ab^	66.3 ± 19.3^ab^	4.85	0.003	0.05(0.90)
Social functioning	77.8 ± 21.2^ac^	84.8 ± 26.0^b^	78.0 ± 20.2^c^	77.6 ± 16.9^abc^	4.28	0.006	0.05(0.86)
School functioning	68.5 ± 24.5	75.0 ± 29.5	71.6 ± 23.2	74.8 ± 19.7	1.97	0.120	0.02(0.50)
HRQOL total score	72.6 ± 15.5^ac^	80.2 ± 18.9^b^	74.6 ± 14.2^c^	76.8 ± 12.4^abc^	7.39	< 0.001	0.08(0.98)

Note: Analysis of covariance (ANCOVA) test was performed and parental educational level, mother’s working status and father’s job were adjusted in the model. Adjusted mean and standard deviation have been reported. ^a-c^: Results of Bonferroni’s post test have been indicated as superscript letters in each row. Means in a row without a common superscript letter significantly differ. * Partial Eta squared is reported as effect size

Table 5 indicates the HRQoL scores according to participant’s satisfaction regarding their body weight using the general linear model (ANCOVA). In girls, except for physical functioning, the subscales and total scores of HRQoL were significantly different according to girls’ satisfaction regarding their body weight. Results of the post hoc test showed that those girls who were satisfied with their body weight had significantly higher scores in emotional functioning (*p* = 0.005), social functioning (*p* = 0.007), school functioning (*p* = 0.004) and total HRQoL (p = 0.004) compared to girls who were dissatisfied with their body weight. In addition, those girls who were satisfied with their body weight had significantly higher social functioning scores (p = 0.007) compared to their counterparts who were neither satisfied nor dissatisfied of their body weight. In boys, only physical functioning, social functioing and total HRQoL scores were significantly different among different groups of adolescents’ satisfaction regarding their body weight. Findings of the post hoc test indicated that boys who were satisfied with their body weight had significantly higher physical functioning (*p* < 0.001) and total HRQoL (*p* = 0.003) scores compared to boys who were dissatisfied with their body weight.

**Table 5:** Adolescent self-reported HRQoL scores according to adolescents’ satisfaction regarding their body weight

	Satisfied	Neither satisfied nor dissatisfied	Dissatisfied	F value	*p*-value	Effect size (power)*
Girls						
Physical functioning	75.9 ± 21.2	75.5 ± 15.9	74.3 ± 19.5	0.39	0.675	0.003(0.11)
Emotional functioning	63.1 ± 31.1^a^	52.9 ± 23.3^ab^	55.0 ± 28.1^b^	6.54	0.002	0.045(0.91)
Social functioning	86.1 ± 23.7^a^	75.7 ± 17.5^b^	80.4 ± 20.6^bc^	8.07	< 0.001	0.055(0.96)
School functioning	73.7 ± 26.1^a^	69.1 ± 19.1^ab^	66.7 ± 23.8^b^	5.59	0.004	0.039(0.86)
HRQOL total score	74.9 ± 18.7^a^	69.2 ± 14.3^ab^	69.8 ± 17.3^b^	6.44	0.002	0.044(0.90)
Boys						
Physical functioning	85.2 ± 22.9^a^	83.3 ± 13.6^ab^	77.4 ± 17.8^b^	9.92	< 0.001	0.071(0.98)
Emotional functioning	69.1 ± 32.3	66.2 ± 19.8	63.8 ± 24.9	2.27	0.105	0.017(0.46)
Social functioning	82.9 ± 28.3	82.7 ± 17.0	76.9 ± 22.2	3.77	0.024	0.028(0.69)
School functioning	72.4 ± 32.3	79.2 ± 19.8	71.3 ± 24.9	1.12	0.327	0.009(0.25)
HRQOL total score	78.4 ± 20.2^a^	78.5 ± 12.8^ab^	73.0 ± 16.0^b^	5.80	0.003	0.043(0.87)

Note: Analysis of covariance (ANCOVA) test was performed and parental educational level, mother working status and father’s job were adjusted in the model. Adjusted mean and standard deviation have been reported. ^a-c:^ Results of Bonferroni’s post test have been indicated as superscript letters in each row. Means in a row without a common superscript letter significantly differ. * Partial Eta squared is reported as effect size

## Discussion

This study aimed to investigate whether body weight status, adolescents’ perceptions regarding their body weight status and adolescents’ satisfactions regarding their body weight were associated with HRQoL of boys and girls residing in Isfahan. The findings of this study indicated that less than one third of adolescents (26.7% of girls and 34.5% of boys) were overweight or obese which is less than previous findings reported by Heidari et al. among 12–14 years Iranian adolescents residing in Isfahan (43 and 45% of girls and boys respectively) [27]. A probable reason for higher prevalence of overweight and obesity in boys versus girls, might be related to the “monitoring” behavior of parents which includes “restriction” factor for girls and “pressure to eat” factor for boys [28]. Parents, especially mothers tend to show the more maternal restriction and control over girls` diet but not boys [29] so boys are more likely to eat more calories from main meals and snacks [30]. Moreover, based on existing evidence boys spend more time on television viewing and playing computer games which can result in higher calorie intake and lower physical activity and consequently more chance for gaining excessive weight [31].In the current study, more than one third of adolescents (42.9%) had misperceptions regarding their body weight status with the higher rates in boys compared to girls. These findings are in line with the findings of CASPIAN study conducted on representative sample of Iranian children and adolescents which reported that about 40% of participants misperceived their body image [23]. In addition, according to findings of the current study, more girls were dissatisfied with their body weight compared to boys; recent findings are in agreement with findings from several countries in Europe, Canada, and the USA [32]. In the current study, boys reported better HRQoL compared to girls specifically in physical functioning and emotional functioning. Additionally, in both sexes, the highest and the lowest HRQoL scores were reported in social functioning and emotional functioning respectively. These findings are in line with the previous study conducted among Iranian adolescents residing in Tehran [21].

In terms of body weight status and waist circumference, findings of the current study indicated that HRQoL scores in physical functioning was significantly lower in underweight adolescents compared to other weight status groups. Although, in the current study, underweight adolescents reported lower HRQoL scores in physical functioning compared to their normal weight, overweight and obese counterparts; this finding was supported by a *p*-value of 0.049, small sample size (*n* = 18), small effect size and low power of the test (64%). Therefore, the interpretation of current findings should be done cautiously. Most existing studies reported significant association between overweight and impaired HRQoL in adolescents [6, 7, 21, 33]; however, in line with our findings, there are few studies which did not find any significant association between overweight and HRQoL [10, 11] specifically in girls [22, 34]. These findings indicate that deviation from normal weight either as underweight or overweight/obese can have a negative impact on HRQoL which implying the possibility of U shape association between BMI and HRQoL. As previous studies also reported U shape association between BMI and HRQoL [35] as well as BMI and depression [36]. Additionally, there was an inverse association between waist circumference and social functioning in girls which indicating that higher waist circumference in girls was associated with impaired HRQoL in social dimension. In terms of waist circumference, most of existing studies mainly have focused on general obesity (determined by BMI) and limited evidence exist regarding the association between abdominal obesity (determined by waist circumference) and HRQoL scores in children and adolescents. In line with our finding, Abdel-Aziz et al. indicated that waist circumference significantly affected total QOL score [37]; similarly, in Germany, central obesity was associated with HRQoL and visits to a physician in primary school children [38]. However, another study conducted on children and adolescents who were severely obese did not find any significant association between waist circumference and EQ-5D utility scores [39]. These inconsistencies may be due to differences in social norms and cultural values regarding ideal body weight, body shape and body size in different population which are influenced by geographic residence. Furthermore, different methodologies and sample sizes may also be responsible for the different results found.

In terms of body weight perception, current findings indicated that boys who perceived themselves as underweight had significantly poorer HRQoL in physical functioning, emotional functioning, social functioning compared to those who perceived themselves as normal weight. Boys who perceived themselves overweight also had poorer physical functioning and social functioning compared to boys who perceived themselves as normal weight. Recent finding is supported by the desire of bigger body size in adolescent boys which is influenced by the ideal of muscular body in boys. This is reasonable, as findings of previous studies reported U-shape association between body dissatisfaction and BMI, in other words, both low BMI and high BMI were associated with body dissatisfaction in boys [40–42] and body dissatisfaction was found to be associated with HRQoL [12].

Finally, current findings indicated that body weight dissatisfaction was associated with poorer HRQoL specifically in psychosocial dimensions (emotional functioning, social functioning and school functioning) in adolescent girls, whereas, its association with poor HRQoL in boys was observed in physical and social dimensions. In agreement with our findings, evidence indicating that body dissatisfaction is a risk factor for future depressive mood and low self-esteem in adolescents [43] and it is related to psychological well-being of adolescents. Additionally, another study conducted among Turkish adolescents reported that self-esteem and depression were more related to body satisfaction rather than actual weight or weight perception [44]. This is in agreement with our findings, as our results also indicating that HRQoL is more related to body dissatisfaction rather than actual body weight and weight perception. Body satisfaction is influenced by the discrepancy between an individuals’ perception of his/her own body weight and his/her ideal body weight. Body dissatisfaction may differ due to changing social norms, cultural values and environmental factors (family and peers influences) [41, 45–47]; hence, environmental and socio-cultural factors influencing body dissatisfaction need to be further investigated in future studies as they may contribute to impaired HRQoL in adolescents. As it has been mentioned before, body dissatisfaction is related to depression, isolation, low self-esteem and other psychiatric and medical complications like body dysmorphic disorder (BDD), eating disorders and comorbid eating pathology, more treatment seeking and unnecessary cosmetic surgeries in future life, inmate hospitalization, higher suicide attempts and higher mortality rate [43, 48, 49]. According to literature, body dissatisfaction is strongly related with cultural expectations and media. Therefore, corrective interventions including legislation and new regulation regarding advertisements in websites, magazines and television are recommended. Promotion of education campaigns in society specifically in schools may be another effective attempt to address body dissatisfaction in both underweight and overweight adolescents.

### Strengths and limitations

Recruiting participants from different geographical zones, wide range of age (12–18 years old) and both sexes are among the strength of the current study. The limitations of the current study should also be considered. BMI Z score was used to define obesity, whereas BMI is not an accurate indicator of body fat and consequently healthy body weight. However, including waist circumference in the current study adds to its strength by providing the chance of investigating the association between body fat distribution and body shape with HRQoL. Another limitation of this study is its cross-sectional nature which limits its ability to show the causality of studied variables and HRQoL. Third, given the small size of this sub-sample specifically in underweight group, the interpretation of current findings should be done cautiously. Furthermore, using one simple question for assessment of perception regarding body weight status and body dissatisfaction was another limitation of the current study. Additionally, information regarding pubertal status was not collected in the current study. Considering possible influences of physical and hormonal changes during puberty on body dissatisfaction, it is recommended to consider pubertal status in future research. Besides, there are other factors that may influence HRQoL of adolescents, especially socio-cultural factors which were not included in the current study and should be further investigated in future studies. Exploring a conceptual model using structural equation modeling approach may help better understanding of pattern of direct and indirect association among potential determinants of HRQoL in Iranian adolescents. Finally, selecting study population from Isfahan city, limits the generalizability of current findings to other parts of Iran or other countries.

## Conclusion

In conclusion, perception and dissatisfaction regarding body weight rather than actual body weight status are responsible for impairment of HRQoL in Iranian adolescents. These findings would provide beneficial information for health personnel and policy makers about the possible predictors of health-related quality of life in adolescents. Current results suggest that more attention should be given to body image as an important determinants of HRQoL during early years of life. Body dissatisfaction may provide an important target for health interventions aiming at improving HRQoL in this age group.

## Abbreviations

ANCOVA: Analysis of covariance
BMI: Body mass index
HRQoL: Health-related quality of life
PedsQL: Pediatric quality of life inventory
WHO: World health organization
